# Annexin II represents metastatic potential in clear-cell renal cell carcinoma

**DOI:** 10.1038/sj.bjc.6605128

**Published:** 2009-06-09

**Authors:** Y Ohno, M Izumi, T Kawamura, T Nishimura, K Mukai, M Tachibana

**Affiliations:** 1 Department of Urology, Tokyo Medical University, Tokyo, Japan; 2 Department of Diagnostic Pathology, Tokyo Medical University, Tokyo, Japan; 3 Clinical Proteome Center, Tokyo Medical University, Tokyo, Japan

**Keywords:** Annexin II, metastatic potential, prognostic factor, clear-cell carcinoma, kidney cancer

## Abstract

BACKGROUND: Annexin II (ANX2) is a multi-functional protein involved in cell proliferation and membrane physiology and is related to cancer progression. The purpose of this study was to assess ANX2 expression in clear-cell (cc) renal cell carcinoma (RCC).

METHODS: The ANX2 expression in 18 primary ccRCCs was examined by real-time reverse transcriptase (RT)–PCR and western blot analyses. Furthermore, immunohistochemical study was performed using paraffin section of 154 primary ccRCCs and 24 metastases. The association between ANX2 expression and the clinicopathological factors and prognosis was analysed.

RESULTS: The ANX2 was upregulated at both mRNA and protein levels in 14 of 18 primary ccRCCs. Immunohistochemical analysis showed that ANX2 was positive in 73 (47.4%) of 154 primary ccRCC and in 21 (87.5%) of 24 metastatic tumours. The ANX2 expression in the primary tumours showed significant associations with a higher stage, a higher nuclear grade. In patients without metastasis, the 5-year metastasis-free rate in patients with ANX2-positive tumour was significantly lower than that in those with ANX2-negative tumour (63.0% *vs* 90.1%; *P*<0.0001). Multivariate analysis showed that ANX2 expression is an independent predictor for metastasis.

CONCLUSION: Our findings suggest that ANX2 expression might be a novel predictor of the metastatic potential of ccRCC.

Renal cell carcinomas (RCCs) account for ∼3% of adult tumours. The RCCs comprise a heterogeneous group of tumours with distinct genetic backgrounds and different biological characteristics. The most common subtype of RCC is clear-cell RCC (ccRCC), which represents over 80% of RCCs (Srigley, 2004). The widespread use of non-invasive imaging techniques (ultrasonography, computed tomography and magnetic resonance imaging) for abdominal screening or unrelated disorders has led to a migration towards earlier stages at diagnosis, with increased numbers of asymptomatic small renal tumour being detected. These incidentally detected RCC have a more favourable prognosis than symptomatic RCC (Pantuck *et al*, 2000). The Tumor-Node-Metastasis (TNM) stage and nuclear grade are currently widely accepted prognostic factors (Shuch *et al*, 2006), however, some patients infrequently follow unexpected clinical course such as late recurrence. Thus, the identification of additional prognostic markers more strictly related to the intrinsic biological behaviour of RCC may be helpful to plan postoperative follow-up protocol in individual patient, as well as to prevent the unnecessary use of adjuvant therapy.

Recently, we succeeded in establishing two RCC cell lines, which were derived from a matched pair of a primary RCC and its adrenal metastasis (Ohno *et al*, 2008). Proteomic analysis showed that annexin A2 (ANX2) is one of the proteins that are differentially expressed between these cell lines. The ANX2, a 34–36 kDa protein, belongs to a family of Ca^2+^-dependent phospholipids and membrane-binding proteins. The ANX2 is involved in specific functions that depend on its intracellular localisation, such as DNA synthesis in the nucleus, protein transportation in the cytoplasm. Further, on the cell surface it functions as a receptor for tissue-type plasminogen activator and tenascin-C (Gerke *et al*, 2005). As for the role in cancer, it is reported that overexpression of ANX2 is associated with progression and metastasis in various types of cancer, including brain tumour (Reeves *et al*, 1992), pancreatic cancer (Diaz *et al*, 2004; Esposito *et al*, 2006), gastric cancer (Emoto *et al*, 2001a) and colorectal cancer (Emoto *et al*, 2001b). Recently, an *in vitro* study of rat RCC showed an association between ANX2 overexpression and metastasis (Tanaka *et al*, 2004). In addition, another report showed that ANX2 expression correlated with higher nuclear grade of the tumour and poor clinical outcome, although the study included only 33 cases (Zimmermann *et al*, 2004). These findings imply that ANX2 may be involved in the metastatic process in RCC. Therefore, in this study, we investigated the ANX2 expression in primary tumours in the context of its prognostic significance in RCC and RCC metastases.

## Materials and methods

### Clinical samples

Fresh frozen surgical specimens and formalin-fixed paraffin-embedded tissues were obtained from patients with ccRCC who were treated at the Tokyo Medical University Hospital. The informed consent for the use of the samples was obtained from each patient.

Fresh frozen surgical specimens of primary tumour tissue paired with normal renal cortical tissue were obtained from 18 patients, who were treated between January 2004 and February 2008, at the time of nephrectomy and stored at −80°C until use. A total of 10 patients had T1 tumours, two had T2 tumours and six had T3 tumours. One tumour was graded as 1; eight as grade 2; nine as grade 3 or 4. At the time of diagnosis, seven patients had metastatic disease, and one patient developed metastasis during follow-up. In addition, formalin-fixed paraffin-embedded tissues were obtained from 161 patients who were treated between September 1986 and September 2003, which included 154 primary ccRCCs and 24 metastatic tumours of ccRCCs. The metastatic tissue samples were obtained by surgical resection or tumour biopsy. The sites of metastasis were as follows: bone (*n*=5), lymph node (*n*=2), adrenal gland (*n*=3), skin (*n*=3), lung (*n*=2), brain (*n*=2), parotid gland (*n*=2), thyroid gland (*n*=1), ovary (*n*=1), gall bladder (*n*=1), retroperitoneal tissue (*n*=1) and soft tissue (*n*=1). The samples included 15 matched pairs of primary tumours and their metastases. The mean age of these 161 patients (117 men and 44 women) at the time of diagnosis was 59 years (range 24–81 years). A total of 154 patients had undergone radical nephrectomy at our hospital. Extended lymphadenectomy was not included in the routine procedure of nephrectomy, but regional lymph node dissection was performed if enlarged or palpable lymph nodes were recognised. No patient received irradiation or chemotherapy before surgery. The tumours were staged according to the 1997 TNM staging system: 79 were TNM stage I, 12 were stage II, 28 were stage III and 35 were stage IV. The histological types were determined according to the World Health Organization classification. Nuclear grading was performed according to the Fuhrman's nuclear grading system: 48 tumours were graded as 1; 68 as grade 2; 38 as grade 3 or 4. All patients were followed-up with clinical and radiological examinations at regular intervals. Patients with metastatic disease received interferon-*α* or interleukin-2 therapy. At the last follow-up, 76 patients showed no evidence of disease, 29 patients were alive with metastases, 46 patients had died of cancer and 10 patients had deceased because of other events. The mean follow-up period was 89.9 months (range, 1–261 months).

### Real-time RT–PCR

A total of 18 pairs of primary RCC and normal renal cortex were used in this analysis.

The total RNA from surgically resected tissues was extracted using the Isogen reagent (Nippon Gene, Toyama, Japan) in accordance with the manufacturer's instructions. The sequence of primer sets used was as follows: *ANX2* forward primer, 5′-TGAGCGGGATGCTTTGAAC-3′; *ANX2* reverse primer, 5′-ATCCTGTCTCTGTGCATTGCTG-3′; *β-actin* forward primer, 5′-ATTGCCGACAGGATGCAGA-3′; *β-actin* reverse primer, 5′-GAGTACTTGCGCTCAGGAGGA-3′. The *β-actin* was run in each PCR reaction and used as an internal control. Real-time reverse transcriptase (RT)–PCR was performed using a SmartCycler system (Cephied, Sunnyvale, CA, USA). The RT–PCR was carried out using a one-step SYBR RNA PCR Kit II (Perfect real time; Takara Biomedical, Tokyo, Japan) according to the manufacturer's instructions. The total RT–PCR reaction volume was 25 *μ*l and contained 2 × One Step SYBR RT-RNA PCR buffer4 (containing the dNTP Mixture, Mg^2+^ and SYBR Green I), PrimerScript 1step Enzyme Mix2, 20 ng of sample RNA and 400 nM of each oligonucleotide primer. The initial steps of RT–PCR were performed for 5 min at 42°C followed by a 10 s hold at 95°C. Thereafter, 45 cycles consisting of a 5 s melt at 95°C, followed by a 30 s annealing/extension at 60°C were performed. The denaturation curve was drawn at the end of the reaction to confirm the melting temperature of the specific PCR product. No template (H_2_O) control was included in each reaction run.

The cycle threshold (Ct) value, which is the cycle number at the point where the fluorescence rises above the background noise, was determined using the second derivative method (Luu-The *et al*, 2005). In the second derivative method, the Ct corresponds to the first peak of a second derivative curve. This peak corresponds to the beginning of a log-linear phase. The relative amount of *ANX2* gene transcript normalised to *β-actin* was determined by the difference in their Ct value (ΔCt) (Hamalainen *et al*, 2001).
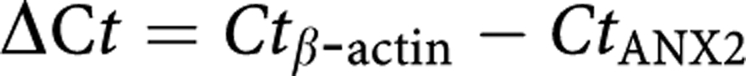


### Western blot analysis

A total of 18 pairs of primary RCC and normal renal cortex were used in this analysis. Whole-tissue protein extracts were collected after sonication with 2 × sample buffer (containing 0.25 M Tris-HCl, 10% 2-Mercaptoethanol, 4% sodium dodecyl sulphate and 10% sucrose). The protein concentration was determined using a Bio-Rad Protein Assay Kit (Bio-Rad Laboratories, Hercules, CA, USA). A total of 10 *μ*g of protein from each sample was run on a 15% sodium dodecyl sulfate–polyacrylamide gel electrophoresis (SDS–PAGE) gel. The proteins were then transferred onto nitrocellulose membranes (Bio-Rad Laboratories) with a Trans-Blot SD Semi-Dry Transfer Electrophoretic Transfer Cell (Bio-Rad). Non-specific binding was blocked with phosphate-buffered saline containing 0.1% Tween 20 and 5% non-fat dried milk for 2 h at room temperature. The membrane was probed with rabbit anti-human annexin II polyclonal antibody (dilution at 1 : 200; Santa Cruz Biotechnology, Santa Cruz, CA, USA) for 1 h at room temperature. The membrane with bound antibodies was then incubated with anti-rabbit horseradish peroxidase-conjugated IgG secondary antibody (dilution at 1 : 1200, Amersham Pharmacia, Piscataway, NJ, USA) for 1 h at 37°C. Immunoreactive proteins were detected with an enhanced chemiluminescence detection kit (Amersham Pharmacia) and LumiVision HS (Taitec Co., Tokyo, Japan). Subsequently, the membranes were stripped off the first probe by using Restore Western Blot Stripping Buffer (Pierce, Rockford, IL, USA) and reprobed with mouse anti-human *β*-actin monoclonal antibody (dilution at 1 : 2000; Sigma-Aldrich, St Louis, MO, USA). The secondary antibody used for anti-human *β*-actin antibody was anti-mouse horseradish peroxidase-conjugated IgG antibody (dilution at 1 : 2000, Amersham Pharmacia). Densitometric analysis was performed by using a LumiVision analyzer (Taitec Co., Tokyo, Japan). The ANX2 protein level was expressed with the relative ratio (RR), which was calculated by the following formula using signal intensity (SI) of ANX2 and *β*-actin.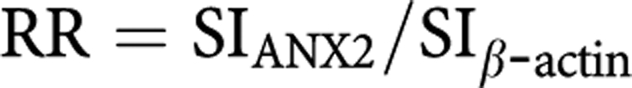 NIH/3T3 whole-cell lysate (sc-2210, Santa Cruz Biotechnology) was used as the positive control.

### Immunohistochemistry

The sections (4-*μ*m-thick) from archival formalin-fixed paraffin-embedded tissues of a representative area of the surgical specimens, which included the highest nuclear grade cancer, were mounted on poly-L-lysine coated slides. They were deparaffinised with xylene and rehydrated through a graded alcohol series. Endogeneous peroxidase was blocked by incubation with 3% hydrogen peroxide for 20 min. Antigen retrieval (121 °C for 10 min in 10 mM citrate buffer, pH 6.0) was then performed. Endogenous biotin was blocked by incubation with 0.01% biotin for 20 min at room temperature. After blocking non-specific conjugation with 1% casein, the slides were incubated for 60 min at room temperature with anti-annexin II rabbit polyclonal antibody (dilution at 1 : 50; Santa Cruz Biotechnology). Bound antibodies were detected by the avidin–biotin complex peroxidase method (Vectastain ABC Kit, Vector Laboratories, Burlingame, CA, USA) and visualised with diaminobenzidine. The slides were counterstained in Harris’ hematoxylin, dehydrated and mounted. Sections of benign prostate tissue were used as positive control. As negative control, the primary antibody was omitted and substituted with normal rabbit IgG.

The ANX2 expression was considered to be positive if >10% of the cancer cells were clearly stained in each slide under microscopic observation.

### Statistical analysis

All statistical analyses were performed using JMP Ver. 5.1 (SAS Institute Inc., Cary, NC, USA). The ANX2 gene by real-time RT–PCR and ANX2 protein expression by western blot were assessed using paired *t*-test. In immunohistochemical analysis, ANX2 expression was considered as a dichotomous variable (i.e., positive or negative) in all the statistical analyses. The correlation between ANX2 expression and other clinicopathological factors was assessed using a χ-square test, Fisher's exact test and Student's *t*-test. The metastasis-free and cancer-specific survival rates were estimated by the Kaplan–Meier method, and the differences between the curves were tested using the log-rank test. Metastasis-free time was calculated from the date of radical nephrectomy to the date of radiological detection of metastases. A multivariate analysis using the Cox proportional hazards regression model was used to test for independent prognostic values. *P*-values <0.05 were considered statistically significant.

## Results

### Annexin II expression in primary RCC tissues: results of real-time RT–PCR and western blot analysis

**Figure 1:**
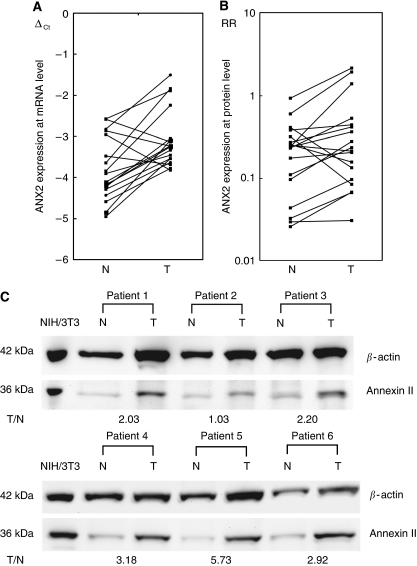
Annexin II (ANX2) expression in primary kidney cancer. (**A**) The *ANX2* expression at mRNA level was analysed in 18 pairs of primary clear-cell renal cell carcinoma (T) and the corresponding normal renal cortex (N). The *ANX2* was significantly upregulated in 14 primary tumours (*P*=0.0086). (**B**) Western blot analysis showed ANX2 expression was higher in 14 of 18 primary tumours compared with the corresponding normal renal cortex; however, the difference was not significant. The relative ratio (RR) is calculated using signal intensity of ANX2 and *β*-actin. (**C**) Representative examples of ANX2 protein expression in primary kidney cancer. After normalising the signal intensity of ANX2, the ANX2 expression in each sample was evaluated on the basis of the ratio of the primary kidney cancer to the corresponding normal renal cortex. Patients 1–3 showed no evidence of metastasis, patient 4 developed metastasis 3 years after nephrectomy, and patients 5 and 6 showed metastasis at the time of diagnosis.

A total of 18 pairs of primary kidney cancer and a corresponding normal renal cortex were analysed. The ANX2 was upregulated at both mRNA and protein levels in 14 of 18 primary RCC tissues in comparison to the corresponding normal renal tissues; especially, the ΔCT value of tumour (mean±s.d., −3.07±0.71) was significantly higher than those of the corresponding normal renal tissue (mean±s.d., −3.80±0.76) (*P*=0.0086) (Figure 1A and B).

Representative examples of ANX2 expression by western blot analysis are shown in Figure 1C. The primary kidney cancer (T)/the corresponding normal renal cortex (N) ratio of ANX2 expression was 0.33–5.73 (mean ratio, 1.96). The mean T/N ratio in patients with metastasis (mean±s.d., 2.62±0.44) was more likely to be higher than that in those without metastasis (mean±s.d., 1.44±0.39) (*P*=0.0617).

### Annexin II expression in paraffin-embedded primary kidney cancer and its metastases: results of immunohistochemistry

**Figure 2:**
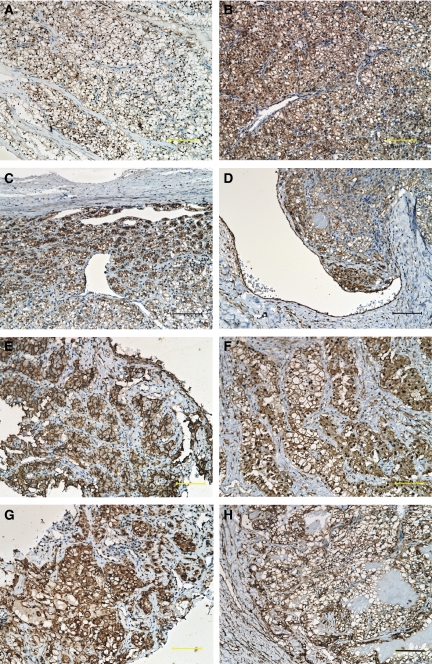
Immunohistochemical staining of ANX2 in primary RCC and its metastases. (**A**) Grade 1 clear-cell carcinoma, (**B**) grade 2 clear-cell carcinoma, (**C**, **D**) ANX2 is highly expressed along the periphery and around vessels. (**E**) Lung metastasis, (**F**) bone metastasis, (**G**) neck lymph node metastasis and (**H**) brain metastasis. Magnification at × 20; Scale bar, 100 *μ*m.

Immunohistochemical analysis of 154 primary RCCs and 24 metastatic tumours was performed. The ANX2 staining was observed on the cell membranes or in the cytoplasm. The ANX2 staining was heterogenous in the tumour sections, and the staining intensity was relatively higher in the periphery of the tumour and around vessels (Figure 2A–D). Finally, 73 (47.4%) of the 154 primary RCCs and 21 (87.5%) of the 24 metastatic tumours were considered to be positive for ANX2. There was a significant statistical difference in ANX2 positivity between a primary RCC and its metastasis (*P*=0.00028). Among the 15 matched pairs of primary RCC and its metastasis, both components were positive for ANX2 in 12 pairs. Representative images of the metastatic tumours (lung, lymph node, bone and brain) are also shown in Figure 2E–H.

### Correlation between annexin II expression and clinicopathological factors

Table 1 shows the correlation between ANX2 expression in primary RCC and clinicopathological factors in the 154 patients who underwent nephrectomy. The ANX2 expression was more frequent in tumours of a higher TNM stage (*P*<0.0001), higher T stage (*P*=0.0006) and higher nuclear grade (*P*<0.0001). The M1 stage (*P*=0.0003) and microscopic venous invasion (*P*=0.007) were also associated with ANX2 positivity.

### Prognostic significance of annexin II expression in RCC

To assess the prognostic significance of ANX2 expression in RCC, we analysed cancer progression (metastasis) and survival in relation to ANX2 expression in primary tumour.

**Figure 3:**
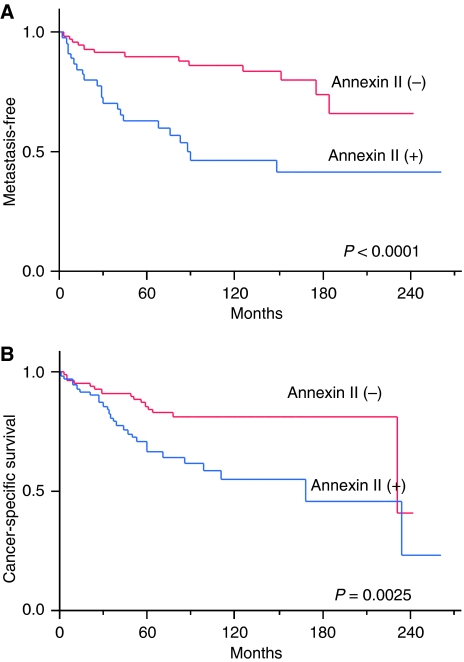
Metastasis-free survival rates in patients with stages 1–3 and the cancer-specific survival rates. The metastasis-free survival rates were estimated by the Kaplan–Meier method, and the differences between curves were tested using the log-rank test. (**A**) The metastasis-free survival rates in patients with stages 1–3 disease (*n*=119). The metastasis-free time was calculated from the date of radical nephrectomy to the date of radiological detection of metastases. The metastasis-free survival rate of patients with ANX2-positive primary tumour was significantly lower than that of patients with ANX2-negative primary tumour (*P*<0.0001). (**B**) Cancer-specific survival rates (*n*=154). The survival rate of patients with ANX2-positive primary tumour was significantly lower than that of patients with ANX2-negative primary tumor (*P*=0.0036).

The relationship between clinicopathological factors and progression was analysed in 119 patients with stages 1–3 disease. A total of 41 patients developed metastases during their clinical course. The median time to radiological appearance of metastases was 50.5 months (range, 1.5–184 months). The following were the first recognised sites of metastasis: the lung in 26 cases, bone in 4 cases, liver in 1 case, adrenal gland in 1 case, retroperitoneal space in 4 cases and others in 5 cases. Metastasis-free rate was estimated using the Kaplan–Meier method. There was a significant inverse correlation between metastasis-free rate and the TNM stage (*P*=0.0003) or nuclear grade (*P*=0.0001) of the tumour. Microscopic venous invasion did not have significant influence on the development of metastasis. As for ANX2 expression, the metastasis-free rate in patients with ANX2-positive primary RCC was 63.0% over 5 years and 46.5% over 10 years; this rate was significantly lower than the 90.1% 5-year metastasis-free survival and 83.6% 10-year metastasis-free survival observed in patients with ANX2-negative primary RCC (*P*<0.0001) (Figure 3). Multivariate analysis with a Cox proportional hazard model showed that ANX2 expression was an independent predictor for metastasis (*P*=0.021; Table 2).

With regard to cancer-specific survival, there were significant differences in TNM stage (*P*<0.0001), nuclear grade (*P*<0.0001), microscopic venous invasion (*P*=0.0011) and ANX2 expression of the primary RCC (*P*=0.0025). However, multivariate analysis showed that only TNM stage and nuclear grade were independent prognostic factors for survival (TNM stage, *P*<0.0001; nuclear grade, *P*=0.0002) (Table 2).

## Discussion

In this study, we investigated the ANX2 expression in primary ccRCCs and their metastases. In agreement with earlier reports (Unwin *et al*, 2003; Domoto *et al*, 2007), *ANX2* expression was upregulated in primary RCC compared with the corresponding normal renal cortex at mRNA and protein levels. Furthermore, western blot analysis showed that ANX2 protein level was more likely to be higher in primary tumours that developed metastases than in those that did not, as described in earlier report of rat RCC model (Tanaka *et al*, 2004). In immunohistochemical study, ANX2 was positive in 73 (47.4%) of the 154 primary ccRCCs and in 21 (87.5%) of the 24 metastatic tumours. The ANX2 positivity in primary tumour was clearly associated with advanced stage and a higher nuclear grade. Univariate and multivariate analysis showed that the immunohistochemical expression of ANX2 in a primary tumour is an independent predictor of metastasis.

The ANX2, a 34–36 kDa protein, is a multi-functional protein involved in cell proliferation and membrane physiology (Gerke *et al*, 2005). The dysregulation of ANX2 expression has been reported in various types of cancers, thus far. Upregulation of ANX2 expression has been reported in hepatocellular carcinoma (Frohlich *et al*, 1990), lung cancer (Cole *et al*, 1992), pancreatic cancer (Diaz *et al*, 2004; Esposito *et al*, 2006), breast cancer (Sharma *et al*, 2006), gastrointestinal cancer (Singh, 2007), glioma (Reeves *et al*, 1992) and colorectal cancer (Emoto *et al*, 2001b). Conversely, downregulation of ANX2 expression has been reported in prostate cancer (Chetcuti *et al*, 2001; Banerjee *et al*, 2003; Liu *et al*, 2003), osteosarcoma (Gillette *et al*, 2004), oesophageal squamous cell carcinoma (SCC) (Zhi *et al*, 2003) and head and neck SCC (Pena-Alonso *et al*, 2008). This discrepancy may be explained by ANX2 expression in the histological origin of tumours, the cellular localisation and the putative function of ANX2 in tumour cells. The ANX2 exists as a monomer in the cell cytoplasm or as a heterotetramer complexed with S100A10 in association with the plasma membrane. The ANX2 heterotetramer acts as a receptor for plasminogen, tissue-type plasminogen activator and tenascin C, and is implicated in extracellular matrix degradation (Gerke *et al*, 2005). The ANX2 is not observed in normal hepatocytes, the pancreatic exocrine system and the mammary gland (Frohlich *et al*, 1990; Dreier *et al*, 1998; Massey-Harroche *et al*, 1998); in tumours that arise from these tissues, such as hepatocellular carcinoma, pancreatic cancer and breast cancer, respectively, ANX2 expression is observed as membrane and/or cytoplasmic staining (Frohlich *et al*, 1990; Diaz *et al*, 2004; Sharma *et al*, 2006). Esposito *et al* (Esposito *et al*, 2006) reported that ANX2 expression changed from cytoplasmic to cell surface expression with progression of pancreatic cancer. Sharma *et al* 2006 also reported that ANX2 was expressed on the cell surface of an invasive/metastatic breast cancer cell line and that ANX2-dependent localised plasmin generation by breast cancer cells could contribute to angiogenesis and metastasis. These reports suggest that membrane-associated ANX2 is involved in the degradation of the extracellular matrix, which is required for tumour invasion. On the other hand, it is reported that ANX2 expression is lost as benign prostatic epithelium progresses to prostate cancer (Chetcuti *et al*, 2001). The ANX2 is normally observed in the prostate gland, wherein it localises in the cytoplasm and intracellular side of the plasma membrane and is involved in tethering cytoskeletal proteins or vesicle transport (Liu *et al*, 2003; Gerke *et al*, 2005). Liu *et al* reported that the re-expression of ANX2 inhibited the migration of prostate cancer cells and that a reduction or loss in ANX2 expression may contribute to prostate cancer development and progression. However, it is also reported that ANX2 is re-expressed in poorly differentiated prostate cancer and that ANX2 expression is observed in the metastatic androgen-unresponsive PC-3 and DU-145 cell lines (Banerjee *et al*, 2003; Yee *et al*, 2007). The ANX2 protein may change its distribution or is re-expressed and plays different roles with tumour progression even in the same tumour.

With regard to RCC, the functions of ANX2 have not been fully elucidated. As reported by Zimmermann *et al*, we also showed that ANX2 was highly expressed in the periphery of the tumour and around the vessels. Furthermore, in our current 15 matched pairs of a primary tumour and corresponding metastatic tumour, both components were positive for ANX2 in 12 pairs. It is speculated that ANX2 might be associated with extracellular matrix degradation and ANX2-positive tumour cell develop metastasis.

Distant metastasis is a major clinical determinant of the outcome of RCC. However, RCCs occasionally follow an unpredictable course involving events such as late recurrence over a decade after nephrectomy. Thus, it is important to identify reliable prognostic markers to establish individualised follow-up protocols. In this study, we showed that ANX2 expression in ccRCC was associated with metastasis and poor prognosis. The ANX2 might play an important role in the development of metastasis and might be a useful marker for formulating individualised follow-up protocols as well as for identifying patients suitable for adjuvant therapy.

## Figures and Tables

**Table 1:** Correlation between annexin II expression and clinicopathological factors

	**Negative (%)**	**Positive (%)**	***P*-value**
Age (years)	57.3	60.1	NS
Size (cm)	5.3	6.9	0.0028
			
*Gender*			
Male	50 (44.6)	62 (55.4)	0.001
Female	31 (73.8)	11 (26.2)	
			
*TNM stage*			
I	58 (73.4)	21 (26.6)	<0.0001
II	4 (33.3)	8 (66.7)	
III	10 (35.7)	18 (64.3)	
IV	9 (25.7)	26 (74.3)	
			
*T stage*			
T1	58 (66.7)	29 (33.3)	0.0006
T2	9 (42.9))	12 (57.1)	
T3	13 (31.7)	28 (68.3)	
T4	1 (20.0)	4 (80.0)	
			
*M stage*			
0	74 (59.7)	50 (40.3)	0.0003
1	7 (23.3)	23 (76.7)	
			
*Grade*			
1	37 (77.1)	11 (22.9)	<0.0001
2	32 (47.1)	36 (52.9)	
3 or 4	12 (31.6)	26 (68.4)	
			
*Microscopic venous invasion*			
Negative	63 (60.0)	42 (40.0)	0.007
Positive	18 (36.7)	31 (63.3)	

**Table 2:** Univariate and multivariate analysis of metastasis-free and cancer-specific survival rate

	**Metastasis free (%)**				
	**5 years**	**10 years**	**Log-rank test**	**Likelihood Ratio Chi-square**	**Cox**
*TNM stage*					
1	89.6	81.7	0.0003	3.2923	0.1928
2	75.0	42.9			
3	52.5	46.7			
					
*Nuclear grade*					
1	88.5	82.9	0.0001	3.9556	0.1384
2	79.5	68.0			
3 or 4	43.3	34.7			
					
*Microscopic venous invasion*					
Negative	81.8	73.1	NS		
Positive	74.1	60.2			
					
*Annexin II expression*					
Negative	90.1	83.6	<0.0001	5.3228	0.021
Positive	63.0	46.5			
					
	**Cancer-specific survival (%)**				
	**5 years**	**10 years**	**Log-rank test**	**Likelihood Ratio Chi-square**	**Cox**
*TNM stage*					
1	94.4	90.4	<0.0001	26.7984	<0.0001
2	91.8	73.3			
3	74.1	63.6			
4	25.2	0.0			
					
*Nuclear grade*					
1	97.6	97.6	<0.0001	17.0858	0.0002
2	81.3	69.0			
3 or 4	42.0	33.7			
					
*Microscopic venous invasion*					
Negative	83.4	77.6	0.0011	0.0748	0.7845
Positive	63.4	53.9			
					
*Annexin II expression*					
Negative	84.2	81.9	0.0025	0.1022	0.7492
Positive	66.5	54.8			
